# Patient perceptions of physiotherapy and prehabilitation before hip and knee arthroplasty in South Africa

**DOI:** 10.4102/ajod.v15i0.1763

**Published:** 2026-04-08

**Authors:** Prithi Pillay-Jayaraman, Allan R. Sekeitto, Verusia Chetty, Stacy Maddocks

**Affiliations:** 1 Physiotherapy Private Practice, Dr. GM Pitje Day Hospital, Johannesburg, South Africa; 2 Department of Physiotherapy, School of Health Sciences, University of KwaZulu-Natal, Durban, South Africa; 3 Department of Orthopaedic Surgery, Faculty of Health Sciences, Charlotte Maxeke Johannesburg Academic Hospital, Johannesburg, South Africa; 4 Division of Orthopaedic Surgery, Faculty of Health Sciences, University of the Witwatersrand, Johannesburg, South Africa

**Keywords:** arthroplasty, physiotherapy, prehabilitation, knowledge, perception

## Abstract

**Background:**

Hip and knee joint arthroplasty in many low- and middle-income countries has lengthy waiting lists as trauma-related procedures are prioritised. A comprehensive care pathway that includes prehabilitation can enhance patient outcomes. Prior to designing an impactful service, a contextually grounded study with an understanding of current knowledge, relationship between felt and expressed needs in the specific socio-cultural setup is critical.

**Objectives:**

This study aimed to investigate patients’ knowledge and perceptions of physiotherapy and prehabilitation before undergoing total hip or knee replacement operations in a South African public healthcare setting.

**Method:**

A mixed-methods approach utilising semi-structured, face-to-face interviews and questionnaires was employed. Patients in the orthopaedic outpatient queue who consented to participate were recruited. Forty-nine participants answered the questionnaires, and seven patients were interviewed. Questionnaire data were analysed descriptively, including estimates of means and percentages and qualitative data by content analysis.

**Results:**

A significant (*p* < 0.001) 77.6% (38) of patients indicated that they were unsure of the role of a physiotherapist, and 67.3% (33) of respondents were unsure of the necessity for pre-operative exercises (*p* < 0.001). Three main themes emerged from the interviews, which were patients’ preconceptions of physiotherapy, the perceived value of prehabilitation and patients’ recommendations regarding physiotherapy and prehabilitation.

**Conclusion:**

This study identified a lack of knowledge and understanding regarding physiotherapy and prehabilitation among these patients.

**Contribution:**

The findings of this study may inform the design of a prehabilitation programme tailored to this context and highlighted a need for the education of medical personnel.

## Introduction

### Background

The World Health Organization (WHO) has identified osteoarthritis (OA) and rheumatoid arthritis (RA) as chronic musculoskeletal conditions projected to increase in prevalence with an ageing population (Woolf & Pfleger 2003). When conservative management of RA and OA is no longer effective and quality of life diminishes, primary total joint arthroplasty (TJA) for the hip and knee is commonly recommended as a successful treatment for advanced OA (Dunn 2012; O’Neill, McCabe & McBeth 2018).

In South Africa and other low- and middle-income countries (LMICs), however, waiting lists for TJA are long, with significant costs involved in managing these patients (Abera Abaerei, Ncayiyana & Levin 2017). As per the statistics kept at a tertiary hospital in Gauteng, in 2024, there were 2089 patients awaiting arthroplasty and the next available theatre booking date was May 2029. Similar waiting times are common across academic and regional hospitals throughout the province (Dunn 2012). These delays stem from limited resources, the high cost of joint replacements and the prioritisation of trauma cases, which restricts bed availability and operating theatre time for elective surgeries. Consequently, patients face prolonged periods of chronic pain and limited mobility. Furthermore, waiting longer than 6 months can negatively impact post-operative satisfaction and patient-reported outcomes 1 year after total knee arthroplasty (TKA) (Desmeules et al. 2009). In a study using the EuroQol five-dimension questionnaire, many patients reported that the wait for an arthroplasty felt ‘worse than death’ (Scott, MacDonald & Howie 2019).

Multiple factors contribute to extensive waiting lists and high costs, including limited application of outcome-based, multidisciplinary, enhanced recovery pathways (Plenge et al. 2018; Swenson et al. 2018). However, there are no current, accepted national or regional policies on TJA or care pathway guidelines that offer recommendations. There are some South African–based studies which suggest that prehabilitation could serve as a key component of enhanced recovery. Studies have shown a link between inpatient length of stay and cost reduction and recommend addressing patient-related factors before surgery (Plenge et al. 2018; Swenson et al. 2018).

In the authors’ experience, socio-cultural ideologies, perceived benefits of physiotherapy, fear of pain and other factors impact post-operative mobilisation and serve as inhibitors to progress and have been studied in this context but in a different population group (Dangor et al. 2021). Other anecdotal factors that may indirectly affect mobilisation include the presence of long-standing unmanaged foot deformities, poor biomechanics, reduced movement efficiency because of back pain, chronic pain in other areas and a lack of understanding regarding lifestyle management. An exploration of these factors was briefly analysed in a scoping review of prehabilitation intervention by the first author (Pillay-Jayaraman, Chetty & Maddocks 2025); however, linkage in this population has not been specifically studied. All these factors can hinder early post-operative mobilisation in this specific setting. Currently, there is no available literature addressing the presence and influence of these factors in this context or in this population group.

Addressing these issues requires careful consideration of the context and needs of patients, as well as the development of a context-specific programme. It is well recognised that public health challenges are often rooted in complex social, political and economic factors (Moffatt et al. 2006). Additionally, empowering patients in their recovery by patient education and improving information-seeking behaviour are suggested as one of the most critical determinants of improved post-arthroplasty outcomes because of the low literacy levels in this cohort (Plenge et al. 2018). Hence, stakeholder input is essential, particularly in South Africa, because of all the above reasons and the fact that practices from high-income countries cannot be implemented as is. Plenge et al. (2018) serve a rationale for this study. As an upper-middle-income country (The World Bank 2022), South Africa provides arthroplasty in both state-funded tertiary centres and private hospitals. However, approaches proven in well-resourced settings may not be applicable because of the unique socioeconomic landscape in South Africa, shaped by a history of inequality impacting numerous sectors including healthcare (Plagerson 2023).

To design an effective programme, it is essential to conduct a needs assessment. This involves identifying the ‘felt need’, or individual perception of healthcare needs, and determining whether these are expressed as demands for healthcare (O’Brien 2010). Thus, a comprehensive understanding of the relationship between felt needs, expressed needs, socio-cultural factors and context is critical (Jinks, Ong & Richardson 2007) for developing an impactful service.

In our study, we explored patients’ existing knowledge and perceptions of physiotherapy and prehabilitation using a mixed-methods approach, enabling a robust, in-depth analysis. Exploring existing knowledge is essential so the programme and its various elements can address the misconceptions, if any, and will also enable the programme to be pitched and built up from the current level of understanding. We also examined patients’ educational needs regarding physiotherapy and prehabilitation, with the aim of informing a tailored prehabilitation programme. This contextually grounded study, we believe, is an essential first step towards implementing programmes that can reduce waiting times and improve patient outcomes. This study is phase two of a larger study, and the protocol has been previously published (Pillay-Jayaraman et al. 2025). Phase one of the study was an extensive literature review which informed elements of the design of this study as well as the questionnaire design and has been published (Pillay-Jayaraman, Chetty & Maddocks 2023). Phase 3 involved comparing standard care pathway to the prehabilitation pathway. The standard care pathway was informally tested, and its efficacy reported in an unpublished manuscript and adopted as the standard pathway in a published manuscript which details the specific intervention and timelines (Ebrahim et al. 2021).

## Research methods and design

A mixed-methods approach was used to assess patients’ understanding of physiotherapy, with a particular focus on the concept of prehabilitation, as well as to explore their perception of physiotherapy approaches.

This approach was chosen for its capacity to provide rigorous, methodologically sound insights, allowing the authors to analyse and integrate data from both qualitative and quantitative streams to strengthen the overall findings (Creswell, Fetters & Ivankova 2004). By employing a triangulation design model, the authors were able to converge comprehensive categories of data to gain a thorough understanding of the research topic (Creswell et al. 2004). The qualitative element complemented and aided in securing a greater depth of understanding of patient’s perspective and allowed exploration and complementing elements that could be missed because of language barriers from the quantitative aspect of the study. Quantitative and qualitative methods and findings are discussed separately, with integrated discussion of results from both streams (Creswell et al. 2004; Schoonenboom & Johnson 2017).

Ethical approval was obtained from the University of KwaZulu-Natal (BREC/00002141/2020) before initiating the study, and informed consent was obtained from all participants. Participation was voluntary, and anonymity was ensured by assigning coded identifiers.

### Study setting and population

Our study was conducted at a large tertiary teaching hospital in Soweto, Johannesburg, South Africa. This hospital, the third largest globally and the largest in the Southern Hemisphere, serves a population of over 3.5 million people with a capacity of 3 200 beds. Patients with hip and knee pain are referred by a general practitioner in a primary healthcare setting if their condition warrants a higher level of care. On arriving at the tertiary hospital, patients are managed by orthopaedic specialists, and information regarding their condition, various aspects of care and primary arthroplasty operation as a treatment of choice is given during one-on-one consultation with the orthopaedic surgeon. Referral to relevant multidisciplinary healthcare professions is done by them who will refer patients as needed. Hence, it is the discretion of an orthopaedic surgeon to refer patients to physiotherapy or other multidisciplinary healthcare practitioners. Patients who had been on the arthroplasty waiting list for over a year and were attending the outpatient clinic were included in the study. Arthroplasty clinics were held twice a week on Tuesday and Thursday between 8:00 and 12:00 for new patients, repeat appointments and post-operative patients, all following the designate queue. Every second patient in the queue who met our inclusion criteria and was reasonably conversant in English was invited to participate. This method was chosen to preserve an element of randomness, as the outpatient arthroplasty clinic was the only place patients congregated. The quantitative prospective, single-centre study took place from January 2022 to May 2022. Data collection beyond this period was not done, as most of the patients had already been recruited, and no new patients consented to be in the study. The length of time to answer the questionnaire and be in the interview could have been a deterrent to further recruitment. In addition, most patients reported to be physically and mentally fatigued, as they had to leave their home early, travel a distance in a taxi, queue for extended periods of time standing to collect their hospital file and then queue to see the orthopaedic surgeon. Most patients were also reliant on family, friends, paid transport to travel to and from the appointment as well as to accompany them because of the extent of disability and distance to access transport and hence could not wait longer than absolutely necessary. Patient recruitment and data collection were managed by an independent researcher who was not a part of the study and who was conversant in the local language, and a total of 49 patients were recruited to answer the questionnaires, while seven patients agreed to in-depth interviews.

Those who consented were taken to a private room where they completed a questionnaire using the Research Electronic Data Capture (REDCap), a secure web-based platform for data collection and storage. The independent researcher facilitated completion on her smartphone and was available to clarify questions as needed.

The questionnaire (see Table 1 and Online Appendix 1) was divided into six sections, and knowledge was assessed with a scoring system: a ‘No Knowledge’ score of 0 was assigned to patients who responded ‘Don’t know’ or left answers blank; a ‘Fair’ score of 1 for 1–2 correct answers; ‘Good’ score of 2 for 3–4 correct answers and ‘Excellent’ score of 3 for all correct responses.

**TABLE 1:** Key question areas in the questionnaire.

Section category	Description
Section 1: Demographic	Age, sex, level of education, occupation, co-morbidities, work, hobbies
	Responses: Numerical, yes, no
Section 2: Living conditions	Type of dwelling, steps, toilet, transport
	Responses: Numerical, yes, no
Section 3: Pain and associated problems	Painful joints, impairments, activity and participation restriction, assistive devices usage, waiting time for operation
	Responses: Numerical, yes, no
Section 4: Knowledge about the operation	Understanding about operation, risks, complications
	Responses: Open-ended and scoring system
Section 5: Knowledge about prehabilitation	Understanding about prehabilitation, exercise knowledge, compliance
	Responses: Yes, no, open-ended and scoring system
Section 6: Knowledge about post-operative rehabilitation	Understanding about rehabilitation, mobilisation expectations, discharge criteria, exercise knowledge, compliance
	Responses: Yes, no, open-ended and scoring system

Data were exported to Excel and SPSS for analysis by a biostatistician. Descriptive data were analysed with percentages, and significance was set at *p* < 0.05. Pearson’s chi-square and Fisher’s exact tests were used to examine correlations. Open-ended responses were coded and organised into thematic categories.

The qualitative component used convenient sampling of seven patients awaiting arthroplasty, and the final number of seven patients was arrived upon because there was saturation of the information being collected. Hence, this was the deciding factor in considering the data collection process as complete, and further recruitment was deemed redundant.

Data were collected through individual, semi-structured interviews (Online Appendix 2), conducted by an experienced researcher fluent in IsiZulu, the patients’ primary language. Interviews were flexible, audio-recorded and later transcribed and translated into English by a professional linguist. Analysis followed the principles of conventional content analysis (Hsieh & Shannon 2005). Prior to coding and analysis, member checking was conducted through post-interview summary validation, whereby participants were provided with a brief summary of their interview responses and key themes shortly after transcription. They were invited to confirm, clarify or correct the interpretations to ensure accuracy and resonance with their intended meaning. This was done to ensure participant validation and to enhance credibility and trustworthiness.

**FIGURE 1:**
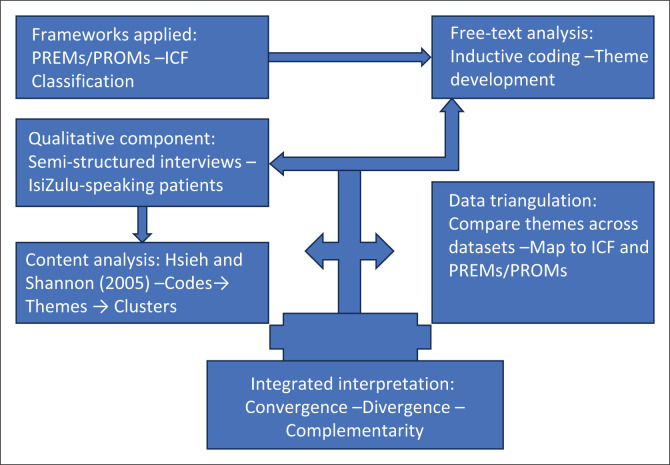
Flow diagram illustrating the integration of qualitative and quantitative data in a convergent mixed-methods design.

The analysis began with repeated reading to achieve immersion and a comprehensive sense of the data. This was followed by a detailed, word-by-word examination to identify key concepts and ideas. Through this process, labels for codes were developed through a conventional content analysis approach, as described by Hsieh and Shannon (2005), whereby coding categories were derived inductively from the data without imposing preconceived categories capturing overarching thoughts. Codes were then organised into themes, reflecting the interrelations and patterns among them, and grouped into meaningful clusters for interpretation. Although coding was conducted inductively following conventional content analysis (Hsieh & Shannon 2005), a deductive element was introduced during the interpretive phase. Emergent themes were subsequently mapped onto established frameworks (International Classification of Function [ICF] and Patient-Reported Outcomes [PROMs]) to guide synthesis and ensure coherence across qualitative and quantitative strands, reflecting a hybrid analytic strategy appropriate to mixed-methods research. A flow diagram illustrating the integration of qualitative and quantitative data in a convergent mixed-methods design is depicted in Figure 1. Frameworks were applied to guide analysis and synthesis, with triangulation used to compare themes and enhance interpretive validity.

### Ethical considerations

Ethical clearance to conduct this study was obtained from the University of KwaZulu-Natal and the Biomedical Research Ethics Committee on 16 August 2022 (No. BREC/00002141/2020).

## Results

The following section is a breakdown of results from the quantitative questionnaire responses. Of the 49 arthroplasty patients who participated in the surveys, 83.7% (41) were female, ranging from 40 years to 80 years old; 32.7% (17) were aged between 51 years and 60 years, followed by 31.6% (14) between 61 years and 70 years; 24.5% (12) of the sample were employed; 38.8% (19) were unemployed; 34.7% (17) were pensioners; 2% (1) received a disability grant; 63.3% (31) of the respondents had not completed secondary school. Additional demographic information is summarised in Table 2 and further information is available in Table 1-A3, Online Appendix 3.

**TABLE 2:** Replacements required by sample.

Question	*n*	Operation	Months
**What operation are you waiting for**			
	10	THR R	-
	12	THR L	-
	15	TKR R	-
	12	TKR L	-
**How long have you waited for the operation**			
	3	-	0–11
	11	-	12–24
	13	-	25–36
	22	-	≥ 37

THR R, total hip replacement right; THR L, total hip replacement left; TKR R, total knee replacement right; TKR L, total knee replacement left.

### Knowledge of physiotherapy

Knowledge of physiotherapy, patients’ exposure and prior referral to physiotherapy was assessed by open- and closed-ended questions. While 100% of the respondents had been managed with pain medication, only 14.2% (7) were referred for physiotherapy.

**FIGURE 2:**
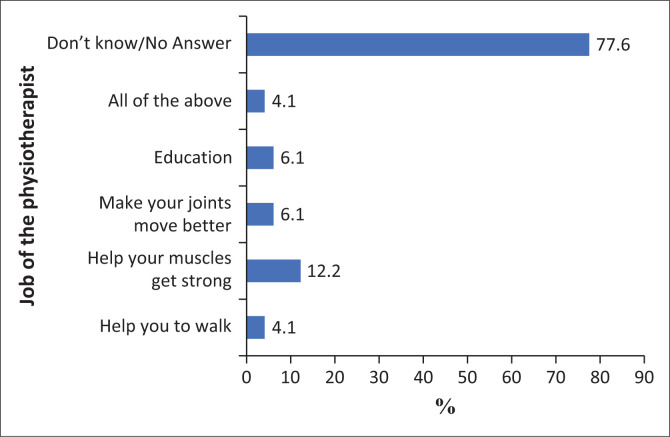
Knowledge of the role of physiotherapist.

In response to the question on the role of a physiotherapist (Figure 2), a significant (*p* < 0.001) 77.6% (38) of patients indicated that they were unsure of the role or function of a physiotherapist. Pearson’s Chi-square and Fisher’s exact test was conducted to ascertain the relationship between this knowledge and education level (Table 3: Educational level of the sample), that is, completed matric/not completed matric, and it was found that no significant relationship existed with *p* = 0.240.

**TABLE 3:** Educational level of the sample.

Category	Frequency	%
Did not complete matric	31	63.3
Matric	12	24.5
Diploma	5	10.2
Graduate	1	2.0

### Knowledge of prehabilitation

A significant 67.3% (33) of respondents were unsure of the necessity for pre-operative exercises (*p* < 0.001). Only 8.2% believed exercises should not be done, while 24.5% (12) indicated that pre-operative exercises were necessary.

When asked about the type of information they would have liked regarding prehabilitation from the open-ended questions, patients expressed a need for guidance on exercise types, exercise indications and precautions, visual examples of positive patient outcomes and instructions on assistive device use. Specific areas of interest included exercises for muscle strengthening, gait improvement, pain relief and stiffness prevention. One patient noted:

‘The one that will help me reduce weight and make my muscles strong enough to withstand the operation.’ (I8)

Patients also enquired if physiotherapy could have prevented the need for surgery and expressed concerns about exercise safety and its impact on pain. One patient remarked:

‘What exercises do I do without making it [*referring to pain*] worse and damaging my joint.’ (VV22)

Some respondents, however, stated that they would not have done exercises because of pain, and a few reported being advised by community clinic physiotherapists to avoid exercises until after surgery.

### Compliance with prehabilitation

When asked if they would perform exercises pre-operatively if the need was explained, an overwhelming 93.9% (46) of patients indicated they would be willing to participate (*p* < 0.001). Additionally, 88% (43) reported that receiving an exercise pamphlet would possibly motivate them to exercise (*p* < 0.001), and 80% (39) felt that SMS reminders would encourage them to continue with exercise (*p* < 0.001).

However, 90% of respondents indicated that attending face-to-face therapy once a week was not feasible. Reasons for non-attendance included cost and transportation challenges. Open-ended responses highlighted the financial impact on family members who would need to accompany them, resulting in lost workdays and wages. Patients reported difficulties using public transport because of limited accessibility and expressed discomfort in navigating these spaces with mobility aids. One patient explained:

‘Can’t use a taxi as it’s too painful, and the drivers and passengers react negatively if I take too long.’ (H7)

Another mentioned:

‘Taxis leave you and don’t stop if they see you have crutches.’ (G6)

### Knowledge of rehabilitation

Regarding post-operative exercise timing, the unit’s practice recommends (Pillay-Jayaraman et al. 2025) beginning exercises in bed within a few hours after surgery. However, as shown in Table 4, a significant 83.6% of patients were unaware of this recommendation (*p* < 0.001).

**TABLE 4:** Knowledge of rehabilitation.

Responses	How long after the operation can you exercise?		How long after the operation can you walk?	
	*n*	%	*n*	%
Few hours after the procedure	2	4.1	1	2.0
The day after the procedure	6	12.2	6	12.2
Two days after the procedure	13	26.5	15	30.6
A week after the procedure	13	26.5	10	20.4
Unsure	15	30.6	17	34.7

**Total**	**49**	**100.0**	**49**	**100.0**

In response to the question about when patients could start walking post-operation, only 12.2% (see Table 4) were aware that mobilisation could begin the day after surgery. However, a significant 85.7% were unaware of the timing for walking post-surgery, a statistically significant finding (*p* < 0.001).

**FIGURE 3:**
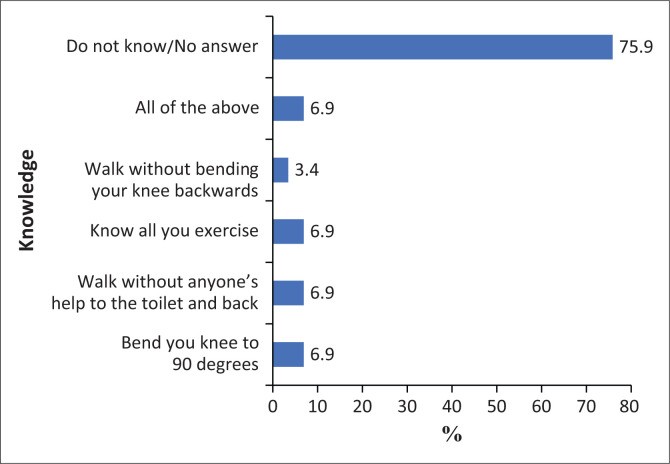
Knowledge on discharge criteria of the knee.

Additionally, 75.9% (22) of patients did not know what functional abilities were required before discharge, a result that was also statistically significant (*p* < 0.001, see Figure 3). Specifically, 90% (18/20) of patients awaiting hip replacement were uncertain about the discharge criteria they needed to meet, which again showed statistical significance (*p* < 0.001).

The data collected from the semi-structured in-depth interviews are presented in the following. The demographic information of the seven patients interviewed is summarised in Table 5, and Table 6 contains a summary of the patient characteristics and baseline knowledge. The names contained herewith are pseudonyms. Participants’ ages ranged from 55 years to 78 years, with most having waited over 3 years for their surgery. Notably, most participants had not completed their matriculation and presented with associated comorbid conditions.

**TABLE 5:** Patient information.

Patient	Sex	Age (years)	Operation	OA classification	Time waiting for operation (years)	Co-morbidities	Level of education
Amelia	F	58	TKR L	Grade 4	3	Asthma and hypertension	Did not complete matric
Bopelo	F	65	THR R	Grade 4	> 3	Hypertension	Did not complete matric
Cathy	F	61	TKR L	Grade 4	> 3	Hypertension	Did not complete matric
Demakatso	F	78	THR L	Grade 4	> 3	Hypertension	Did not complete matric
Emily	F	55	THR R	Grade 4	> 3	Hypertension	Did not complete matric
Florah	F	67	TKR R	Grade 4	> 3	Hypertension	Did not complete matric
Gugu	F	61	TKR L	Grade 3	1	Hypertension	Did not complete matric

F, female; OA, osteoarthritis; THR R, total hip replacement right; THR L, total hip replacement left; TKR R, total knee replacement right; TKR L, total knee replacement left.

**TABLE 6:** Patient characteristics and baseline knowledge.

Patient	Level of function	Preparation for the operation	Understanding about physiotherapy	Knowledge of the operation and source	Reason to have the operation
Amelia	Independent with self-care but struggled with bending her knees and speed of walking. Domestic worker, so is always late to work.	By being brave and following what the healthcare professional’s advice after the operation.	Hear about physiotherapy from family. Has not had physiotherapy, not referred.	Not educated, so does not understand much about the operation. Saw the information on the TV in the waiting area.	‘It will help me with walking.’ ‘I will walk normal again without the pain.’
Bopelo	In a lot of pain, so cannot sit, stand for a long time, cannot go outside. Cannot iron anymore or use a bathtub. Faced a lot of unkindnesses from people when travelling.	Got exercises from a co-worker who felt sorry for her and made her a book of exercises and a plastic (TheraBand).	Does not know about physiotherapy. Has not had physiotherapy, not referred.	Knows about a ball and they will put iron. Watched on TV but forgot. Doctor also spoke about it.	‘I want my legs to be the same length, so I can be able to walk.’ ‘I want to be like other people.’
Cathy	Can do some tasks like cooking, but cannot do laundry or cultivate (gardening: used to grow spinach, etc., to use in the house).	She knows she has to exercise after the operation, and it will help her heal fast after the operation.	Not referred for physiotherapy before the operation and has not had physiotherapy.	She knows that her knee is damaged and the doctor can fix it. ‘They put in the bones. Got the information from the TV in waiting area, doctor and another patient who had the operation.’	Fix the damaged knee.
Demakatso	She is able to do some things but has constant pain even at night.	‘Hope and positivity, I need to have hope that everything will do well.’	‘I had heard about it; they said it will help after the operation, but you must also play your part, and you need to follow every instruction that they gave you so that the healing process will be quick.’	She forgot what they said about the operation, but she knows the problem will be fixed.	She can move freely and not be in pain.
Emily	‘I can do most things but I have a house at Bram-fisher, I can’t go there unless they take me with a car. My house roof is leaking I can’t fix it on my own because I can’t climb the ladder.’	‘I should exercise so I can be fit, and I should make sure that my muscles are flexible.’	‘It will help me with speedy recovery.’ Not referred for physiotherapy.	‘I only know that they are going to insert … eish what do they call it? Mmm a new what what, they will insert it then after that I should not do anything. I should relax for a short period of time until it gets better; it heals.’ ‘Knowledge from healthcare professionals.’	Tired of using crutches and the pain.
Florah	‘I try to do most of the work, but I take breaks in between; I work, sit down and work again like that, but I cannot go on my knees anymore, use a stick and its very painful.’	Did not comment about this despite prompting.	‘I once did it some time ago. I removed my womb; after that, I had to do exercises with them.’	‘Mmmh, it is a knee replacement operation; means they are going to insert an iron so it can replace the bones that are not functioning properly.’ Doctor and TV.	Relieve the pain and improve the way she walks.
Gugu	Cannot walk well; that is the problem.	‘I spoke to friends, and they said will be no after-effects; I must just listen to the doctor, therapists and do everything that is asked for, and I will be fine.’	‘Daughter is a doctor, so she took me to a physiotherapist and did some exercises.’	Going to have a replacement of the knee, do not have much more information. ‘I prayed to God and I know I will get better.’	‘I cannot manage with the pain anymore and cannot stand for a long time and walk. So, I need to have the operation.’

### Emerging themes from patient interviews: Physiotherapy and prehabilitation

Three main themes were identified during analysis: patients’ preconceptions of physiotherapy, the perceived value of prehabilitation and patients’ recommendations for physiotherapy and prehabilitation. While these thematic areas were broadly informed by the interview guide, the specific content and subthemes were inductively derived from participants’ narratives, consistent with a conventional content analysis approach.

### Patients’ preconceptions of physiotherapy

Participants exhibited uncertainty regarding the nature and scope of physiotherapy. For instance a 78-year-old woman awaiting a total hip replacement, remarked:

‘I don’t know physiotherapy, but I have heard about it. I think all this [*physiotherapy*] is about exercising.’ (Demakatso)

Many patients derived their understanding of physiotherapy from the experiences of family and friends. For example, a woman also awaiting a total knee replacement, recounted:

‘I first saw it [*physiotherapy*] when my sister was very sick because of diabetes and spent months in the ICU. We even thought she was no longer with us. When she finally woke up, she started physiotherapy in a rehabilitation centre. She did not recognise any of us initially, but after the physiotherapy, her memory returned.’ (Amelia)

Five patients based their understanding of physiotherapy on their experiential knowledge of the profession and their expectations of health benefits. They believed that physiotherapy would facilitate improved mobility, expedite healing and enhance recovery post-surgery. A woman awaiting a total knee replacement, stated:

‘Let me say it helps by teaching us what we are supposed to do after the operation at home so that the recovery can proceed more quickly.’ (Cathy)

### Perceived value of prehabilitation

The theme of perceived value of prehabilitation highlighted that most patients lacked awareness of prehabilitation and its role in preparing them for surgical intervention. This lack of knowledge may stem from their absence of prior experiences with physiotherapy, either personally or through their social circles. One patient expressed this uncertainty:

‘Not very sure about how and what to do’, referring to prehabilitation.’ (Amelia)

One patient with prior exposure to prehabilitation noted that, while the programme did not alleviate her pain, the exercises helped prevent joint stiffness. A woman awaiting a total hip replacement, said:

‘He showed me some exercises, but I could not do them all. When I attempted the exercises, it did not relieve the pain, but it ensured my leg did not become stiff. It was sore to do the exercises.’ (Bopelo)

Similarly, other participants reported experiencing benefits in range of motion as a result of prehabilitation. A 61-year-old woman awaiting a total knee replacement, stated:

‘The exercises before the operation did help, so my knee is bending nicely and did not become stiff.’ (Gugu)

### Patients’ recommendations for prehabilitation

The final theme identified was patients’ recommendations regarding essential information that should be included in a prehabilitation programme, particularly about surgical procedures and post-rehabilitative care. Generally, patients expressed a desire for detailed information about their options and how best to prepare for surgery. The patient articulated this need:

‘I would like them to tell me exactly what I need to do before the operation. I want something that will be helpful, because without the information, you cannot make informed choices. So, I want something that will guide me through this entire process.’ (Amelia)

Patients also expressed eagerness to understand the implications of engaging in prehabilitation versus not participating. Another woman awaiting a total knee replacement, asked:

‘I would like to know if I do the exercises before and after the operation, what will be the difference? If I have the operation without going to a physiotherapist, what will happen? And what will happen if I do go to the physiotherapist beforehand?’ (Florah)

Moreover, patients highlighted the importance of general health management, including weight and fitness, for surgical preparation and post-operative recovery. The patient further expressed:

‘What I would like to know is how I can manage my weight gain. If someone could recommend exercises to address my weight, that would be helpful. I would also like physiotherapy professionals to provide me with exercises to do at home after I’m discharged from the hospital so I can maintain my fitness.’ (Amelia)

## Discussion

To our knowledge, few studies have assessed patients’ understanding of physiotherapy and prehabilitation in resource-constrained upper-middle or lower-middle-income countries, particularly among those awaiting arthroplasty. A novel aspect of this study is its mixed-methods approach through triangulation, which enhanced the rigour of the findings and facilitated the identification of gaps in knowledge. For example, while qualitative interviews have not revealed a lack of knowledge about physiotherapy among medical officers, the quantitative data indicated only a minimal referral rate to physiotherapy, highlighting the value of our employing a mixed-methods strategy. Our data revealed that referrals for physiotherapy were inadequate, with a notable emphasis on pharmacological management to address pain. This reliance on pharmacology as a primary means of pain management has been similarly documented in the literature (Jinks et al. 2007; Porcheret et al. 2007). Physiotherapists have various approaches to manage pain including electrotherapy and musculoskeletal techniques; however, the lack of knowledge of such rehabilitation approaches by assessing multidisciplinary team members may result in a lack of referral to this cadre of healthcare professionals (Crawford, Miller & Block 2013; Kon et al. 2012; Zhang et al. 2008).

This finding is significant because pharmacological management does not promote joint loading and has minimal potential to alter joint function, which is crucial in the pathogenesis of OA (Crawford et al. 2013; Kon et al. 2012; Zhang et al. 2008). Moreover, pharmacological treatments carry substantial risks and financial costs. Therefore, physiotherapy and other interventions that offer safe and effective alternatives should be actively pursued as clinical adjuncts in managing these patients (Crawford et al. 2013; Kon et al. 2012; Zhang et al. 2008). A scoping review of the literature on the topic of prehabilitation by the authors also concluded that prehabilitation could be a valuable adjunct in reducing length of hospital stay and improving functional outcomes in adults undergoing total joint replacement (Pillay-Jayaraman et al. 2023). Despite existing literature advocating for a multifaceted approach to OA management, the continued dependence on pharmacology warrants further investigation to address this issue effectively. Additionally, the implications of using similar pharmacological agents pre-operatively and postoperatively in resource-constrained public healthcare settings, where there may be limited pharmaceutical options, require more in-depth analysis.

The qualitative data from patients in this study highlighted a significant lack of understanding regarding physiotherapy, which limited the authors’ ability to gather recommendations for a prehabilitation programme. In contrast, the quantitative interviews clarified specific areas of knowledge that patients sought regarding prehabilitation, such as exercise types, precautions, outcome-based instructional videos and the use of assistive devices. This information is critical in informing the design of an effective prehabilitation programme. Our findings indicated that patients were predominantly unaware of prehabilitation, with many believing that exercising before surgery could damage their joints or exacerbate pain. This belief is similar to the findings of Wilcox et al. (2006), who identified pain, fatigue, mobility issues, fear and perceived negative outcomes as barriers to participation in prehabilitation programmes. Similarly, Lambert et al. (2000) noted that fear of pain and joint damage prevented patients from engaging in prehabilitation prior to arthroplasty. In contrast, Grime, Richardson and Ong (2010) found that some patients did not fear joint damage and believed that exercise could help maintain their function and independence. Despite this, fear of pain and joint damage emerged as dominant beliefs hindering engagement in prehabilitation, highlighting the urgent need for educational interventions to enhance patients’ understanding of the benefits and risks of such programmes.

Not only is a prehabilitation programme essential for patient care, but it also plays a pivotal role in the decision-making process. In a qualitative study by Mann and Gooberman-Hill (2011), patients reported feeling ill-informed and anxious about making decisions regarding surgery, resulting in a sense of loss of control. This finding is similar to our study, where patients expressed a desire for information to empower their decision-making regarding arthroplasty surgery.

Regarding knowledge sources, our findings are similar to those of Hall et al. (2008) and Hawker et al. (2004), who identified acquaintances, friends, family members and medical personnel as primary sources of information. Parsons, Godfrey and Jester (2009) also noted that potential patients learned from each other’s lived experiences and coping strategies. Given the important role of friends and family in the decision-making process, these results promote the potential need for well-organised support groups as integral components of any prehabilitation programme. Furthermore, Hawker et al. (2004) emphasised the importance of medical personnel as information sources, with almost all participants considering their family physicians critical for health information. Thus, the findings of our study suggest that educating medical personnel about physiotherapy and prehabilitation services should be a priority in service planning.

Another important aspect highlighted by our study was the lack of patient inquiries regarding the surgical process or the acute post-operative phase. We revealed that participants lacked knowledge about physiotherapy management, exercise prescriptions and discharge criteria for their orthopaedic conditions. These knowledge gaps corroborate findings from other studies (Mann & Gooberman-Hill 2011; Soever et al. 2010) that emphasise the necessity of pre-operative education for optimal patient care and recovery. Many participants did not ask about expected pain levels or activity limitations following surgery, nor did they seek information on the nature and extent of rehabilitation required. Instead, their focus was primarily on the anticipated surgical outcomes. These results are similar to the findings of Hall et al. (2008), who noted that their participants also lacked this knowledge or motivation to seek it. They recommended focusing on acute post-operative outcomes during pre-operative preparations, as reduced activity and increased pain can significantly impact length of stay and overall patient experiences and outcomes. We concur that a lack of preparation regarding rehabilitation outcomes leaves patients unprepared for the intense rehabilitation and mobilisation demands post-surgery. Especially if patients are unaware when they should mobilise and the need to exercise, then it will affect outcomes, impact length of stay and reduce satisfaction. Therefore, educational sessions in a pre-operative optimisation programme should encompass acute care expectations and the recovery trajectory. This highlights the necessity of prehabilitation programmes that include specific elements to facilitate pre-operative optimisation, thereby preventing pre-operative disability and enhancing post-operative recovery (Hall et al. 2008).

In addition to addressing prehabilitation and post-operative rehabilitation, Parsons et al. (2009) identified a lack of general support and information related to managing OA symptoms for individuals awaiting hip and knee replacement surgery. Participants noted that advice and information about the surgical procedure, health maintenance, exercise, walking aids, weight control and symptom management were often inadequate or entirely absent. These findings support our study, in which patients expressed a desire for guidance on weight management. The absence of consistent, healthcare professional-led education or information sessions has hindered patients’ access to necessary support (Parsons et al. 2009). Thus, our results highlight the importance of incorporating diverse topics into prehabilitation programmes.

In summary, the educational needs of adults undergoing total hip and knee replacement surgery should encompass a broad range of topics, confirming the importance of providing comprehensive information packages regarding the procedures. Such information is essential for dispelling misconceptions and enhancing participation and compliance in exercise programmes, which are crucial for optimising patient outcomes. Our study indicates a strong desire for readily available information throughout all phases of the continuum of care, including pre-operative, acute and rehabilitative stages.

We conclude that in addition to focusing on the content of the information provided, the methods and timing of its delivery should be optimised. We recommend employing multiple strategies to improve access to information and therapy, such as pamphlets, web-based resources, tele-rehabilitation and podcasts. These approaches can enhance information availability and accessibility, addressing cost barriers that often hinder patient engagement in therapy. This will help improve compliance and ensure that patients receive adequate sessions to achieve meaningful treatment effects.

### Limitations

Although a limitation of qualitative small group studies is the reduced transferability of results to other contexts, this article employed a mixed-methods approach, which we believe strengthened our findings. A few questions in the questionnaire and method of implementation could have led to responders giving socially desirable answers. The study also presents sampling and recruitment bias, whereby recruiting every second patient in the queue maintained an element of quasi-randomness but still skewed representation. The results have limited generalisability, wherein findings from a tertiary hospital may not reflect broader trends across other regions or healthcare settings, especially rural or private sectors. Patients’ physical and mental fatigue, long travel times and reliance on others may have deterred participation, especially among those with more severe disability or socioeconomic constraints. The implementation of the questionnaires in English only limited participation and is an inherent bias. However, the study serves as a baseline feasibility investigation.

### Recommendations

In order to ensure generalisability, it is recommended that the study be expanded to a multi-centre, multi-health system health frameworks to get equitable representation across different race, language and socioeconomic groups.

## Conclusion

A pre-operative preparation strategy focusing on education, exercise and support groups appears to be a plausible approach to prehabilitation in our study context, enabling a proactive stance in our rehabilitative efforts (Mann & Gooberman-Hill 2011). In addition, this study has provided preliminary data which, if expanded to include a multicultural context and different healthcare settings, would provide robust addendums to a contextually relevant pathway of care. This is an essential precursor to centralised databases and the establishment of a National Joint Registry, which will serve in the best interests of the patient, the country and place a spotlight on quality, efficiency and cost-effectiveness. It is also recommended that more research be done on content and provision of standardisation of information on various aspects of this particular surgery from every member of the multidisciplinary team to ensure optimal gains to patients. Such information should be translated into all South African languages, and research should be done to assess its uptake and efficacy.
